# COVID-19 progression towards ARDS: a genome wide study reveals host factors underlying critical COVID-19

**DOI:** 10.5808/gi.22080

**Published:** 2023-06-30

**Authors:** Shama Mujawar, Gayatri Patil, Srushti Suthar, Tanuja Shendkar, Vaishnavi Gangadhar

**Affiliations:** MIT School of Bioengineering Sciences and Research, MIT-Art, Design and Technology University, Loni Kalbhor, Pune 412201, India

**Keywords:** acute respiratory distress syndrome, COVID-19, JAK-STAT pathway

## Abstract

Coronavirus disease 2019 (COVID-19) is a viral infection produced by the severe acute respiratory syndrome coronavirus 2 (SARS-CoV-2) virus epidemic, which was declared a global pandemic in March 2020. The World Health Organization has recorded around 43.3 billion cases and 59.4 million casualties to date, posing a severe threat to global health. Severe COVID-19 indicates viral pneumonia caused by the SARS-CoV-2 infections, which can induce fatal consequences, including acute respiratory distress syndrome (ARDS). The purpose of this research is to better understand the COVID-19 and ARDS pathways, as well as to find targeted single nucleotide polymorphism. To accomplish this, we retrieved over 100 patients’ samples from the Sequence Read Archive, National Center for Biotechnology Information. These sequences were processed through the Galaxy server next generation sequencing pipeline for variant analysis and then visualized in the Integrative Genomics Viewer, and performed statistical analysis using t-tests and Bonferroni correction, where six major genes were identified as *DNAH7*, *CLUAP1*, *PPA2*, *PAPSS1*, *TLR4*, and *IFITM3*. Furthermore, a complete understanding of the genomes of COVID-19-related ARDS will aid in the early identification and treatment of target proteins. Finally, the discovery of novel therapeutics based on discovered proteins can assist to slow the progression of ARDS and lower fatality rates.

## Introduction

The coronavirus disease 2019 (COVID-19) epidemic began in Wuhan, China, and quickly spread over the world, causing the World Health Organization (WHO) to proclaim a global pandemic on March 11, 2020 [1]. Before symptoms appear, this illness spreads through coughing and sneezing by symptomatic patients and asymptomatic carriers [1]. According to the WHO, there have been 638,175,811 cases, of which 6.6 million deaths have been reported as of November 2022 [2]. This virus infects the respiratory system, which is the most common problem associated with COVID-19, causing dyspnea and acute respiratory symptoms that can develop to refractory pulmonary failure [3]. Acute respiratory distress syndrome (ARDS), a life-threatening form of respiratory failure, is frequent among COVID-19 patients. According to weighted averages generated from several studies with COVID-19 data, ~33% of hospitalised patients had ARDS, affecting about three-fourth ~75% of the COVID-19 patients admitted to the intensive care unit and ~45% of those cases were lethal [3,4]. Berlin criteria state that COVID-19–induced ARDS is significantly different from ARDS caused by other reasons, and as a result, it requires a different approach to care [3,5]. COVID-19 ARDS takes 8 to 12 days to appear, whereas the Berlin ARDS criteria restricts the onset period to one week [3,6]. Individuals with COVID-19 ARDS may have normal or even high lung compliance, which is not the case with patients who have traditional ARDS requiring [3]. COVID-19 separates the severity of ARDS into three groups based on specificity: mild, mild-moderate, and moderate-severe [3,6].

Due to the obvious heterogeneity of ARDS, identifying effective treatments becomes difficult [7,8]. Patients with various types of ARDS have undergone several therapies. In cases with typical ARDS, recruitment exercises, high-dose corticosteroids, and ongoing neuromuscular blocking medications are the most often employed adjuvant therapy [3]. Numerous studies have been conducted, and they all refer to ventilator management using mechanical ventilation as the most effective treatments [9]. Mechanical ventilation works on limiting the tidal volume and pressure and maintaining positive end expiratory pressure [7-9]. The use of appropriate therapeutics combined with a pharmacological approach was another method for treating ARDS. Due to its anti-inflammatory properties from both a preventive and a therapeutic perspective, corticosteroids are currently the subject of numerous clinical trials testing their efficacy in treating ARDS [3,9,10]. Other clinical trials using therapeutics like statins, heparin, and aspirin are also ongoing [10]. Utilizing stem cells to restore damaged tissue and creating 3D models of the lungs, alveoli, and bronchial tree to test treatments are two further modern scientific approaches [10]. Given the limited number of effective treatments for established ARDS, ARDS research has shifted its strategic focus to identifying patients with or at high risk of ARDS early in the course of the underlying illness, when strategies to prevent ARDS and associated organ failures can be systematically evaluated. Patients with severe and critical COVID-19 were highly advised to get systemic corticosteroid therapy, however those with non-severe COVID-19 were not [2,3]. Although there are presently no proven effective treatments for COVID-19, a number of medications are being tested in clinical research, including lopinavir-ritonavir, remdesivir, ruxolitinib, and tocilizumab [3,11]. Despite significant advances in our knowledge and treatment of ARDS patients, it still has a high morbidity and mortality rate [9,10,12].

Other than the mechanical and therapeutic approaches, genomic studies are going on for better prognosis and treatment of ARDS. The identification of the virus's genome for its various strains has been made possible by the wide use of whole genome sequencing. Genome analysis studies have aided in the identification of COVID-19 transmission and progression to ARDS with the details of the genes involved in the process. Biomarkers are used to understand the molecular pathways and pathology of the disease by analyzing the variants in the genetic variations [13]. Later, with the use of sequence analysis, after numerous researches, we were able to learn about the phylogenetic links between the virus strains as well as other genomic variation and mutation that occurred in it [14]. Next-generation sequencing (NGS) was used to determine the expression characteristics of genes in different organ systems and particular cells after infection with the virus [15]. Eight genes were found to be linked to the molecular pathway in a study of 1,778 infected cases that included quality control and the variants were identified and mapped to their appropriate genes, chromosomes, and associated medical information [16]. In this study, 26 single nucleotide polymorphisms (SNPs) in genes and 12 unique candidates with biological function or strong supporting evidence were discovered. pointing to a high likelihood of participation in altering the clinical phenotype of COVID-19 [17].

The goal of this paper is to understand the progression of COVID-19 to ARDS and to identify probable genes associated in the pathway for which SNP variation analysis is performed on sample sequences to locate SNPs. We worked with 100 samples that were obtained from National Centre for Biotechnology Information (NCBI)’s Sequence Read Archive (SRA) sequences. The data consisted of RNA sequences obtained from patient reverse transcription polymerase chain reaction tests in the Texas, USA region. These sequences were further processed using the Galaxy server pipeline, which offers a wide range of tools for quality control, SNP analysis, and annotation in one location. The pipeline is described further in the paper, along with the tools utilised and the findings. The SNPs were then mapped in Integrative Genomics Viewer (IGV) to identify probable genes and chromosomes implicated in the pathophysiology of COVID-19 and the ARDS pathway. In our study, we also aim to perform statistical analysis. The null hypothesis we formulated stated that these genes do not contribute to the progression of the disease. Our analysis involved conducting t-tests on the data obtained from various samples [18].

## Methods

### Pathway analysis

**Fig. 1.**
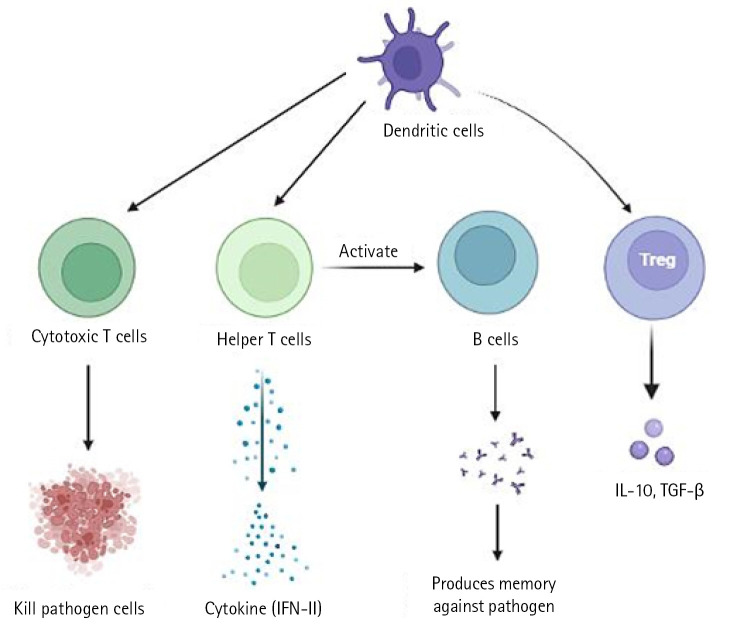
Immunopathology of cells. IFN, interferon; IL-10, interleukin 10; TGF-β, transforming growth factor β.

In a severe acute respiratory syndrome coronavirus (SARS-CoV) animal model, it has been demonstrated that type I interferon (IFN) dysregulation and inflammatory monocyte macrophages result in fatal pneumonia. Therefore, elevated type I IFN secretion and infiltrating myeloid cells have a detrimental effect on the course of the infection and are important contributors to lung damage and function. Dendritic cells deliver pathogen antigens to T cells in lymph nodes, facilitating the cooperation of the innate and adaptive immune systems (Fig. 1).

**Fig. 2.**
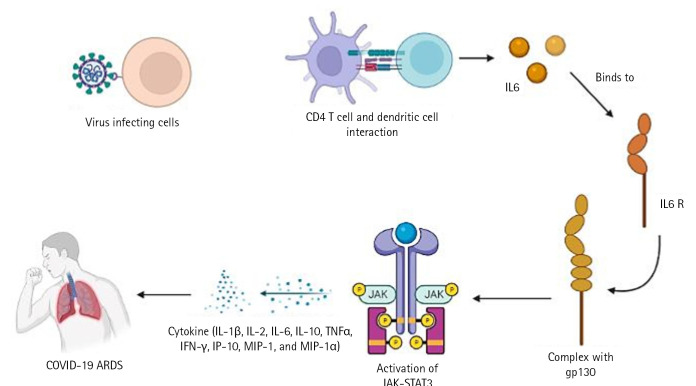
COVID-19–ARDS pathway. COVID-19, coronavirus disease 2019; ARDS, acute respiratory distress syndrome; IL, interleukin; TNF-α, tumor necrosis factor α; IFN, interferon; IP-10, interferon-gamma-inducible protein 10; MIP-1, macrophage inflammatory protein 1.

The cytotoxic T cells recognise the infection and kill the pathogen-infected cells as a result. On the other side, helper T cells trigger IFN-II synthesis. also develops a pathogen memory and stimulates B cells, enabling them to specialise and make pathogen antibodies [19]. Production of interleukin (IL)-10, transforming growth factor, and Treg (regulatory T cell) cells reduces inflammation and re-establishes homeostasis. According to study, CD4+ T cells that produce IL-10 are more prevalent in ARDS patients. In a small proportion of infected individuals, these immune reactions can completely stop viral replication or eradicate virus infection. Others describe a partial viral suppression, a decline in the number of B and T cells in circulation, and then an unknown mechanism. Some people's ARDS caused by COVID-19 has been connected to cytokine storm, a risky complication brought on by protracted viral replication. Some of the cytokines and chemokines that were overexpressed during the cytokine storm were IL-1, IL-2, IL-6, IL-10, tumor necrosis factor, IFN-γ, IFN-gamma-inducible protein 10, and macrophage inflammatory protein 1. Serum IL-6 levels have been associated with the severity of illness and mortality, suggesting a crucial role for IL-6. As IL-6 circulates, it binds to soluble IL-6 receptors and creates a complex with a gp130 dimer on the surface of certain cells. The complex stimulates JAK-STAT3 in a variety of cell types, including endothelial cells, in a subgroup of hospitalised COVID-19 patients, resulting in a cytokine storm and potentially fatal symptoms like ARDS (Fig. 2) [9,20].

### Data collection

The paired Illumina RNA data (Illumina, San Diego, CA, USA) was collected in FASTQ format from the NCBI [21]. Considering that SARS-COVID-19 data is produced using NGS technology, the NCBI SRA includes integrated information [22]. We retrieved a total of 100 SRA sequences in fastq.gz format from Texas, USA, for this objective in preventing variability from various locations, which assisted in limiting within the memory constraint of a large dataset (Supplementary Table 1).

Below filters were applied to collect data:

– Source: RNA

– Type: Genome

– Library layout: Paired

– Platform: Illumina

– File type: Fastq

**Fig. 3.**
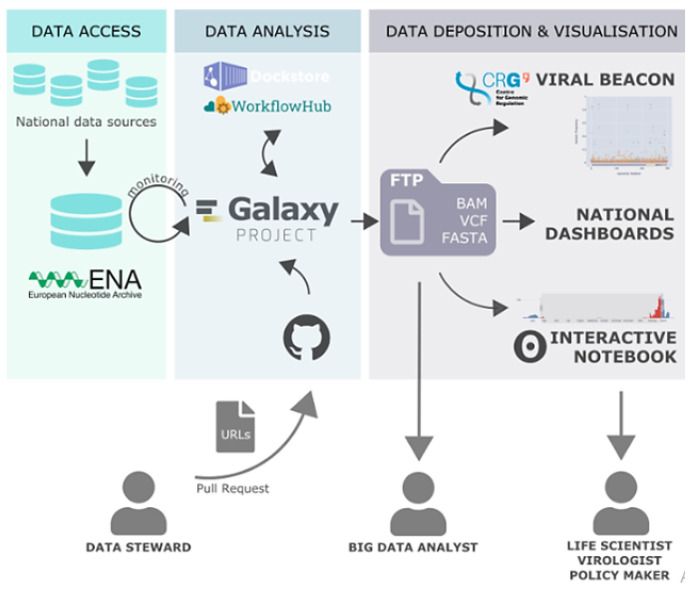
Exploring and monitoring coronavirus disease 2019 (COVID-19) variants with Galaxy.

Since the data was large in number, we made 5 datasets of 20 SRR runs in fastq.gz format for Variant analysis. Additionally, genetic changes (variants) between healthy and diseased tissue, individuals in a population, or strains of an organism can provide mechanistic insight into disease processes and the natural function of impacted genes (Fig. 3) [23].

### Workflow for SNP analysis

SNP analysis was done using Galaxy server [24]. It is a web-based platform to perform analysis of NGS data. Galaxy allows for the characterization, analysis, and computational visualization of genetic data with comparatively minimum resources. Galaxy platform is widely used for developing pipeline for NGS data analysis because of its comparatively high level of accessibility, reproducibility, transparency, and scalability. Galaxy analysis interface has numerous potent tools to perform bioinformatics analysis [25].

**Fig. 4.**
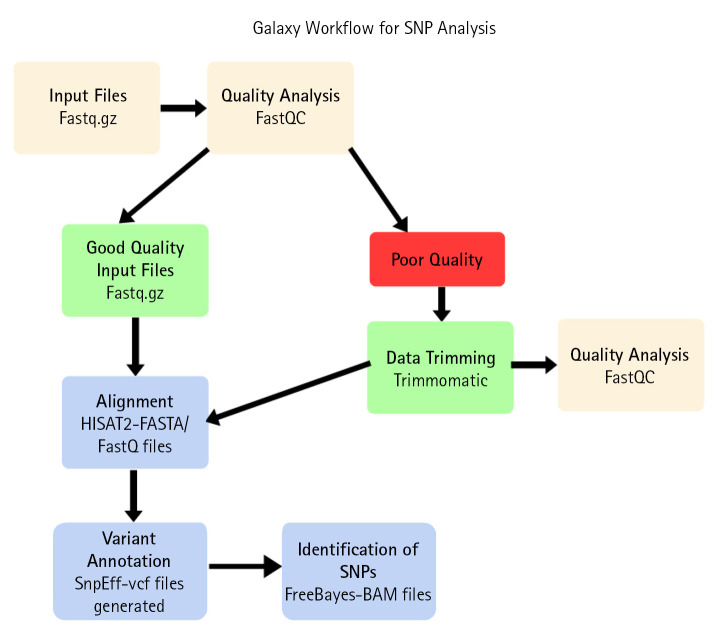
Galaxy server pipeline for single nucleotide polymorphism (SNP) variant analysis.

Therefore, Galaxy server is used for variant analysis. Data downloaded from NCBI, firstly uploaded on Galaxy server. A pipeline for SNP analysis was developed using various tools in the Galaxy server. Different tools used for SNP analysis are FastQC for quality check and analysis, Trimmomatic tool used for trimming reads, SRA sequence aligned with reference human hg38 sequence using HISAT2 tool. SNPs were identified using the FreeBayes tool. Variants annotated by snpEff tool (Fig. 4).

### Quality analysis

Millions of sequences are produced in a single run by modern high-throughput sequencing technologies like Illumina. Therefore, a quality control check is required as the initial step before using raw data for analysis. It is done to determine whether the data is good or if there are any issues with the data. Galaxy has a FastQC (Galaxy version 0.73+galaxy0) tool for quality check of Fastq files. It is a highly effective and popular tool for quality analysis. FastQC analyses fastq files and produces HTML file as an output which can be viewed in Galaxy workspace. The quality report generated gives information about per base sequence quality, sequence content, N and GC content number of reads and their length [26].

Therefore, the FastQC tool was used to perform quality analysis of data. The downloaded SRA runs (fastq.gz) were added to history in Galaxy. FastQC Read Quality reports (Galaxy version 0.73+galaxy0) this tool was selected from the tool menu on the left side. Then the datasets were uploaded in it and executed. The output of FastQC tool contains basic text and HTML file containing information about Basic Statistics, Per base sequence quality, Per sequence quality scores, Per base sequence content, Per base GC content, Per sequence GC content, Per base N content, Sequence Length Distribution, Sequence Duplication Levels, Overrepresented sequences, Kmer Content.

### Data cleaning

FastQC report generates per base sequence quality graph, it shows quality of reads and it is found that in some dataset samples failed in quality checks. A number of the quality measures utilised by FastQC were not met by several of the samples. However, this does not imply that samples should be discarded. Some quality indicators frequently fail, and this may or may not cause issues for your downstream application. To lower false-positive rate owing to sequencing errors, it is required to eliminate some of the low-quality sequences from variant calling method.

To remove low-quality reads and bases from samples, Galaxy has a tool called Trimmomatic (Galaxy version 0.38.0). Trimmomatic is a read trimming tool for Illumina NGS data. It is a flexible tool providing several functions to be operated on reads. These functions include trailing, leading, and several other quality control operations. After performing Trimmomatic on poor quality data, made a dataset collection of good quality SRR reads and trimmomatic output of poor-quality reads.

### Alignment

Alignment of sequences is done to identify the origin of reads. The most time-consuming process in analysis of RNA sequences is alignment of reads against reference genome. So, for fast alignment with less usage of memory of the system, HISAT2 A fast and sensitive alignment program (Galaxy version 2.2.1+galaxy0) tool is used. HISAT2 is hierarchical indexing for spliced alignment of transcripts. A whole-genome Ferragina-Manzini (FM) index is used by HISAT to anchor each alignment, and several local FM indexes are used to quickly extend these alignments. This indexing method is based on the Burrows-Wheeler transform and the FM index [27]. The reference genome used is the Homo sapiens (human) genome assembly GRCh38 (hg38).

### Identification of SNPs

After alignment, the next step is to find out SNPs and MNPs i.e., single nucleotide polymorphism and multi-nucleotide polymorphisms, respectively. The efficient tool available at Galaxy server for SNP identification is Freebayes (version 1.3.1, Galaxy version 1.3.1). It can also determine insertion and deletions i.e., indels and substitutions in the dataset.

Freebayes (version 1.3.1, Galaxy version 1.3.1) will generate a VCF dataset representing SNPs, indels, and complex variations in samples in the input alignments given some BAM dataset(s) and a reference sequence. From the left side tool menu bar, Freebayes is selected and Bam dataset uploaded. The reference genome used is the Homo sapiens (human) genome assembly GRCh38 (hg38) and executed. The variants were identified in .VCF files. The output of Freebayes was given to the next step.

### SNP analysis

These analyzed variants need to be annotated. Variants are changes in the sequence to be analyzed when compared with the reference genome. For annotation Galaxy has SnpEff eff: annotate variants (Galaxy version 4.3+T.galaxy) tool. Variant Call Format (VCF) file is given as input to this tool which has predicted variants. SnpEff eff tool performs annotation of variants and also determines the effects of these variants. Annotation is the process of describing variant information like whether these variants have effect on protein coding or they are present in gene, exon, etc.

VCF file obtained from FreeBayes tool uploaded in SnpEff eff tool. SnpEff Genome version Name used was hg38. Annotation output selected was Use gene ID instead of gene name (VCF output).

### Genome visualization

The IGV (version 2.12.3) is practical, quick, and easy to use software. The IGV is frequently employed for the visualization of genomic data. The real-time visualization of various genomic data sets is made possible by IGV. IGV has the benefit of operating efficiently on a regular desktop computer while consuming the least number of resources. IGV is particularly suited for genome-wide examination of NGS data sets due to its scalable architecture. This high-performance viewer provides a seamless and simple user experience while handling large heterogeneous datasets efficiently. A significant aspect of IGV is its emphasis on the integrative aspect of genomic research, with support for data from both array-based and NGS technologies as well as the blending of clinical and phenotypic information [28,29].

Steps followed for visualization are firstly, IGV was downloaded on desktop. 6 ‘.vcf’ files were uploaded in the IGV. The reference genome that we used is the *Homo sapiens* (human) genome assembly GRCh38 (hg38). Then SNPs were visualized by varying resolution scales.

### Statistical analysis

We applied the Bonferroni correction method to adjust the p-values obtained from the t-tests. This correction is a rigorous statistical technique that reduces the likelihood of type 1 errors by accounting for multiple comparisons. By implementing the Bonferroni correction, we aimed to ensure the reliability and validity of our results by minimizing the possibility of falsely identifying genes as significant contributors to the progression of ARDS.

The t-test was chosen as our statistical tool for several reasons. Firstly, our data met the assumptions of a parametric test, meaning that the distribution of the data after appropriate cleaning and preprocessing was approximately normal. Additionally, the t-test accommodates situations where the variance of the population is unknown, making it a suitable choice for our analysis.

## Results and Discussion

The SNPs, which involve modifications to a single DNA building block, are the most prevalent form of genetic variation in people. They can be applied to study drug response variability and locate disease-causing genes. This has significant implications for the development of genome-based diets and safer drugs as well as personalised treatment. SNPs can also aid in the study of sequence evolution and the underlying biological processes, particularly the involvement of selective forces in human disease.

### Quality analysis

FastQC performed on each read individually and generated output reports were analyzed. Some reads had a good quality score (above 20) lying in the green zone while some had poor quality (less than 20) which has outliers or some part of the sequence in the red zone. These files contained Sanger/Illumina 1.9 encoding of quality values. The Per base sequence quality were analyzed using following criteria:

(1) For each position a Box-Whisker type plot is drawn.

(2) The median value is represented by the central red line.

(3) The inter-quartile range (25%–75%) is represented by the yellow box.

(4) The upper and lower whiskers correspond to the 10% and 90% points, respectively.

The y-axis displays the Phred quality scores. The better the base call the greater the score. The graphical background divides the y axis into calls of extremely high quality (green), calls of reasonable quality (orange), and calls of bad quality (red). Most platforms deteriorate call quality as the run proceeds, so it is normal to see base calls fall into the orange area near the end of a read [26].

**Fig. 5.**
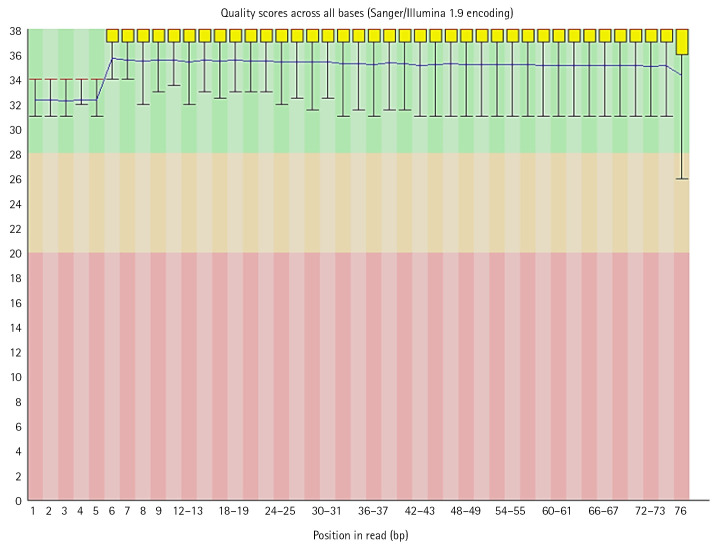
Per base quality score graph.

**Fig. 6.**
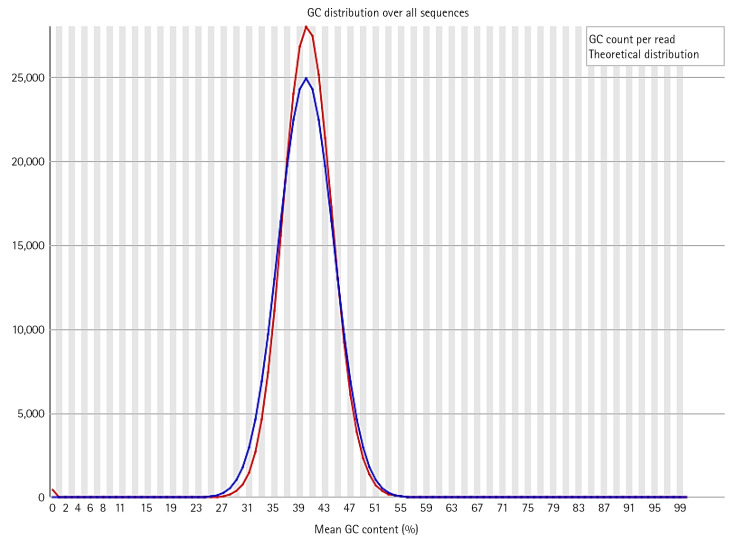
Per sequence GC content analysis.

In the dataset, 50%–60% of the sequences were of acceptable quality, while the remainder were of poor quality. The overall percentage of GC of all bases in all sequences was in between 39 to 44. Along with per base sequence quality, FastQC also gives series of analysis modules. These modules were analyzed for each sequence respectively (Figs. 5, 6).

### Data cleaning

Reads which have poor quality are required to clean. Trimmomatic cleans the data by trimming and performing quality control operations. Poor quality reads can lead to false positive results and can generate errors while running the pipeline. As it can be seen in below images, Trimmomatic improved base quality score.

**Fig. 7.**
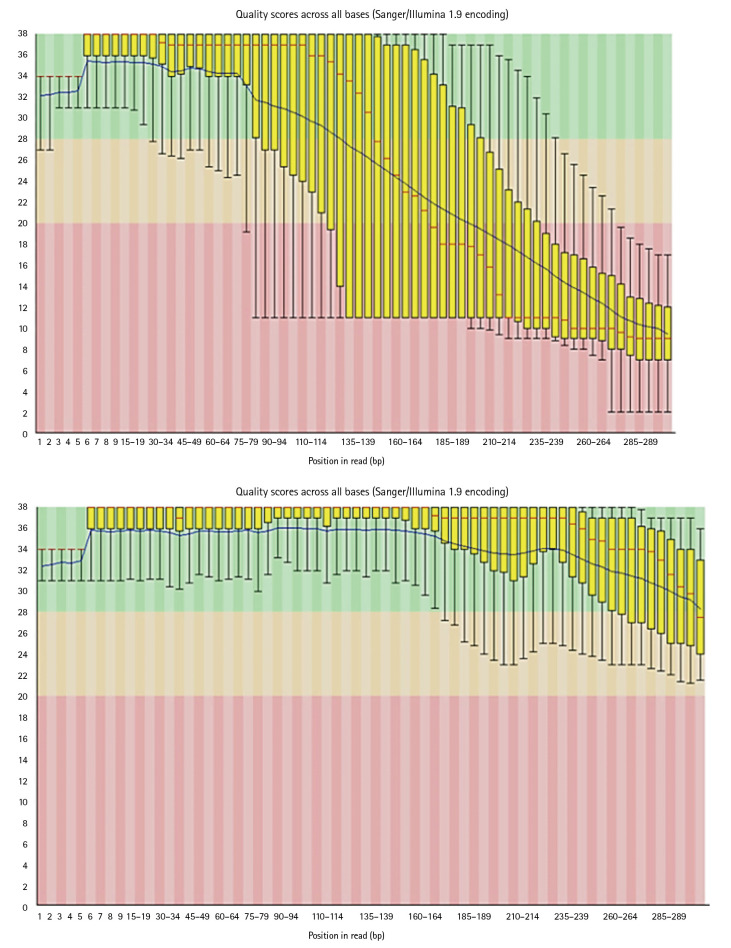
Per base sequence quality before and after trimmomatic.

After trimming low-quality sequences with the Trimmomatic Tool, the sequences quality was verified again with FastQC. The increased FRED quality score generates more precise findings (Fig. 7).

### Alignment

For each single end read file, HISAT2 output generated one bam file. These files are BAM files (short for Binary Alignment Map) and like the name suggests, is a binary file. Galaxy automatically converts these to a plain-text equivalent (SAM) file to view when click on the eye icon. HISAT2 also outputs some information to stderr which we can preview by clicking on the dataset name. The alignment summary was analyzed successfully for the further SNP analysis.

### Identification of SNPs

The BAM file datasets were created for the variant calling. With the correct human genome (Hg 38) reference genome and run-in batch mode with merged output VCFs created the VCFs files for the datasets respectively. Examined the number of lines in the datasets, which is listed in the green box in the History for variants that are exactly in list [30].

### SNP analysis

The default input and output formats for SnpEff are VCF. Since VCF is a common format that may be utilised by other tools and software programmes, it is strongly advised to use it as both an input and output format. Thus, integrating pipelines for analyzing genomic data is much facilitated by VCF.

**Fig. 8.**
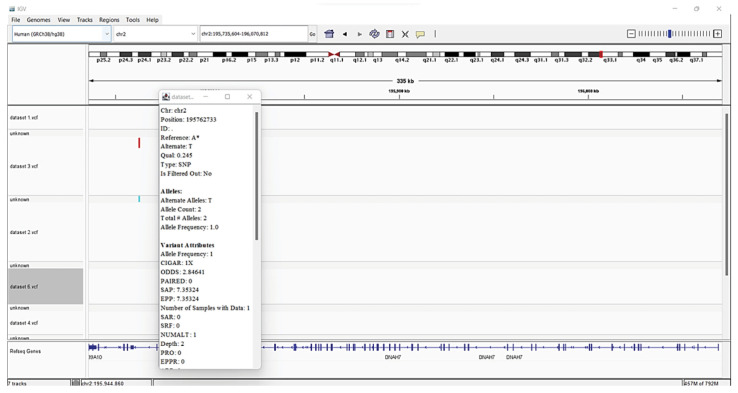
*DNAH7* genes having one mutation.

The identified variations were annotated with information from well-known reference source i.e., Human genome (Hg38). Annotation data is added by SnpEff to the VCF file's INFO field. The eighth column of a VCF file is the INFO field. A summary of the annotations made to the observed variants is included in an HTML document that SnpEff also created. The summaries of the various variant types, their effects, functional zones, etc., were analyzed. Examined tables and graphs and comprehended their meaning [31,32]. The 5 VCFs files were downloaded and viewed in IGV (Fig. 8).

### Genes with SNPs related to COVID-19

The potential drug targets are listed with the help of IGV and were found significant based on the statistical analysis are listed in Table 1. We viewed listed genes related to COVID-19 from the literature and checked for variants in IGV.

There are several SNPs found in chromosome 2 encodes *DNAH7* (dynein axonemal heavy chain 7) gene. The *DNAH7* gene, which codes for dynein axonemal heavy chain 7, a part of the inner dynein arm of ciliary axonemes. After SARS-CoV-2 infection of human bronchial epithelial cells, the gene DNAH7 is reported to be the most downregulated gene, suggesting a potential role in respiratory function. Reduced respiratory cilia performance may result from DNAH7 downregulation. COVID-19 patients who have mutations in *DNAH7* may be more likely to pass away from the disease. The variant is composed of the single SNP, which is found in the promotor region of the gene CLUAP1 (clusterin associated protein 1)'s 5′ untranslated region. *CLUAP1* at chr16_4 also has cilia-related functions. *CLUAP1*, which is produced by the gene CLUAP1, is an evolutionarily conserved protein necessary for ciliogenesis [16]. The severity of COVID-19 was linked to a genetic variation of the interferon-induced transmembrane protein 3 (*IFITM3*) gene, specifically the single-nucleotide polymorphism rs12252. It has been discovered as a potential factor in the susceptibility and severity of respiratory viral infections, including COVID-19, and it may play a role in the pathogenesis of COVID-19 ARDS. Asian groups frequently carry this genetic variation, and influenza severity has been linked to homozygosity for the C allele [33]. There are other two significant SNPs associated with genes related to COVID-19: rs35258888 for the *PPA2* (pyrophosphatase 2) gene and rs70947091 for the *PAPSS1* (3'-phosphoadenosine 5'-phosphosulfate synthase 1) gene, on the other hand, has been linked to an elevated risk of ARDS. A strong correlation between the *PPA2* gene and sudden cardiac death (SCD) has been discovered. Some COVID-19 patients, according to recent reports, had SCD, which ultimately caused their tragic deaths. Reverse transcriptase-polymerase chain reaction analysis of nasopharyngeal swabs and radiological examinations were used in research to identify three COVID-19 patients in July 2020. Ultimately, SCD took these victims' lives. A fair association between SCD and COVID-19 is suggested by the examination of the most recent data, notwithstanding the lack of a direct causal link between the two [34]. TLR4 is a part of a toll-like receptor family, which is associated with the pattern recognition family. TLR4 is a membrane protein, which identifies pathogenic proteins and produces interferons and proinflammatory cytokines in order to fight the infection. In the study done by [35], it was proposed that binding of TLR4 with the viral spike protein led to expression of angiotensin converting enzyme 2 resulting in decrease of air-tissue surface tension and blocking TLR4 thus promoting ARDS. Although the recent studies mention to have a very small or negligible role of TLR4 SNPs resulting in ARDS [36].

### Statistical analysis

In hypothesis testing, a p-value less than 0.05 is conventionally considered statistically significant, leading to the rejection of the null hypothesis. Therefore, if a gene exhibits a p-value less than 0.05, we reject the null hypothesis, indicating evidence to suggest that the gene is associated with the progression of ARDS.

SNPs are variations in the DNA sequence that occur when a single nucleotide (adenine, cytosine, guanine, or thymine) in the genome is changed. Hence, we were able to analyze disease-causing genes in SARS COVID-19 through our research into SNPs, which can lead to ARDS in some situations. This research will aid in the future understanding of medication response diversity among individuals, which has important therapeutic consequences. By demonstrating a link between an individual's genetic make-up and pharmacological response, it may be possible to develop a genome-based diet and drugs that are more effective and safer for each individual. SNPs can also be used to learn about sequence evolution and its molecular mechanisms. The rate, nature, and position of nucleotide changes, as well as the selection pressure on codons, vary across a gene.

It is important to note that rejecting the null hypothesis does not automatically imply acceptance of the alternative hypothesis. Instead, it suggests that we cannot dismiss the possibility of the alternative hypothesis being true. By selecting genes based on their p-values and the associated hypothesis testing, we aimed to identify potential candidates for further investigation and exploration of their involvement in ARDS.

These variations can be used to study the genetic basis of diseases, including COVID-19, and to understand how individuals may respond differently to medications. Research into SNPs and their potential role in disease susceptibility and drug response can help identify genetic markers that may be used to predict an individual's risk of developing a certain disease or their likelihood of responding to a particular medication. This knowledge can be utilised to personalise therapies for each patient and possibly enhance outcomes.

In the case of COVID-19, research into SNPs has identified certain genetic variations that may increase an individual's risk of developing severe ARDS, a serious complication of the disease. This study can contribute to the creation of focused therapies for people who are more vulnerable to ARDS. It is important to note that while SNPs can provide important insights into the genetic basis of disease and drug response, they are just one factor among many that can influence an individual's health. Other factors, such as lifestyle and environmental exposures, also play a role in an individual's overall health and risk of developing certain diseases.

It may be possible to use an individual's genetic makeup to tailor healthcare approaches, including personalized diets and medications that are more effective and safer for them by demonstrating a connection between genetics and pharmacological response.

This has important therapeutic consequences, as it could help to improve the effectiveness of treatments and reduce the risk of adverse reactions. In addition to their potential use in personalized medicine, SNPs can also be used to learn about the molecular mechanisms of sequence evolution. The rate, nature, and position of nucleotide changes, as well as the selection pressure on codons, can vary across a gene. Understanding these processes can provide insights into the evolution of disease-causing genes and may inform the development of new treatments. Overall, the study of SNPs and their role in disease susceptibility and drug response has the potential to significantly advance our understanding of the genetic basis of health and disease, and to improve healthcare outcomes for individuals.

## Figures and Tables

**Table 1.** Variants in identified genes related to COVID-19

Sr. No.	Gene name	Description	Variant Info
1	*DNAH7*	*DNAH7* is involved in cilia dysfunction. Primary ciliary dyskinesia is usually an autosomal recessive genetic condition in which the microscopic organelles (cilia) in the respiratory system have defective function. Ciliary dysfunction prevents the clearance of mucous from the lungs.	Chr 2: 195762733
			SNP; A:T
2	*CLUAP1*	Involved in cilia dysfunction	Chr 16: 3503463
			SNP; A:C
			Chr 16: 3509507-3509510
			MNP: AGGG*:TGGA
3	*PPA2*	Causes myocardial damage in most COVID patients	Chr 4: 105381563
			SNP; T*:C
4	*PAPSS1*	Increases risk of ARDS	Chr 4: 107643163
			SNP; G*:A
5	*TLR4*	Innate immune receptor on the cell surface that recognizes pathogen-associated molecular patterns including viral proteins	Chr 9: 117716883
			SNP; T*:A
6	*IFITM3*	*IFITM3* plays a role in adaptive and innate immune response.	Chr 11: 320115
			SNP; G*:A

COVID-19, coronavirus disease 2019; SNP, single nucleotide polymorphism; MNP, multi-nucleotide polymorphism; ARDS, acute respiratory distress syndrome; DNAH7, dynein axonemal heavy chain 7; CLUAP1, clusterin associated protein 1; PPA2, pyrophosphatase 2; PAPSS1, 3'-phosphoadenosine 5'-phosphosulfate synthase 1; TLR4, toll-like receptor 4; IFITM3, interferon-induced transmembrane protein 3.

## Supplementary Materials

Supplementary Table 1. Total number of patient samples collected from SRA database
