# Supplementary material for: COVID-19 progression towards ARDS: a genome wide study reveals host factors underlying critical COVID-19
Source: Genomics Inform. 2023 Jun 30;21(2):e16. doi: 10.5808/gi.22080 (PMC10326536; doi:10.5808/gi.22080)
Supplement: Supplementary Table 1. — Total number of patient samples collected from SRA database [file gi-22080-Supplementary-Table-1.pdf]

Supplementary Table 1. Total number of patient samples collected from SRA database

Experiment No.	Accession No.	Experiment No.	Accession No.
SRX14416053	SRR18277981	SRX14416074	SRR18277960
SRX14416052	SRR18277982	SRX14416073	SRR18277961
SRX14416051	SRR18277983	SRX14416072	SRR18277962
SRX14416050	SRR18277984	SRX14416071	SRR18277963
SRX14416049	SRR18277985	SRX14416070	SRR18277964
SRX14416048	SRR18277986	SRX14416069	SRR18277965
SRX14416047	SRR18277987	SRX14416068	SRR18277966
SRX14416046	SRR18277988	SRX14416067	SRR18277967
SRX14416045	SRR18277989	SRX14416066	SRR18277968
SRX14416044	SRR18277990	SRX14416065	SRR18277969
SRX14416043	SRR18277991	SRX14416064	SRR18277970
SRX14416042	SRR18277992	SRX14416063	SRR18277971
SRX14416041	SRR18277993	SRX14416062	SRR18277972
SRX14416040	SRR18277994	SRX14416061	SRR18277973
SRX14416039	SRR18277995	SRX14416060	SRR18277974
SRX14416038	SRR18277996	SRX14416059	SRR18277975
SRX14416037	SRR18277997	SRX14416058	SRR18277976
SRX14416036	SRR18277998	SRX14416057	SRR18277977
SRX14416035	SRR18277999	SRX14416056	SRR18277978
SRX14416034	SRR18278000	SRX14416055	SRR18277979
SRX14416033	SRR18278001	SRX14416054	SRR18277980
SRX14416032	SRR18278002	SRX14416009	SRR18278025
SRX14416031	SRR18278003	SRX14416008	SRR18278026
SRX14416030	SRR18278004	SRX14416007	SRR18278027
SRX14416029	SRR18278005	SRX14416006	SRR18278028
SRX14416028	SRR18278006	SRX14416005	SRR18278029
SRX14416027	SRR18278007	SRX14416004	SRR18278030
SRX14416026	SRR18278008	SRX14416003	SRR18278031
SRX14416025	SRR18278009	SRX14416002	SRR18278032
SRX14416024	SRR18278010	SRX14416001	SRR18278033
SRX14416023	SRR18278011	SRX14416000	SRR18278034
SRX14416022	SRR18278012	SRX14415999	SRR18278035
SRX14416021	SRR18278013	SRX14415998	SRR18278036
SRX14416020	SRR18278014	SRX14415997	SRR18278037
SRX14416019	SRR18278015	SRX14415996	SRR18278038

SRX14416018	SRR18278016	SRX14415995	SRR18278039
SRX14416017	SRR18278017	SRX14415994	SRR18278040
SRX14416016	SRR18278018	SRX14415993	SRR18278041
SRX14416014	SRR18278020	SRX14415992	SRR18278042
SRX14416011	SRR18278023	SRX14415991	SRR18278043
SRX14416010	SRR18278024	SRX14415990	SRR18278044
SRX14415989	SRX14415980	SRR18278054	SRR18278045
SRX14415988	SRX14415979	SRR18278055	SRR18278046
SRX14415987	SRX14415978	SRR18278056	SRR18278047
SRX14415986	SRX14415977	SRR18278057	SRR18278048
SRX14415985	SRX14415976	SRR18278058	SRR18278049
SRX14415984	SRX14415975	SRR18278059	SRR18278050
SRX14415983	SRX14415974	SRR18278060	SRR18278051
SRX14415982	SRX14415973	SRR18278061	SRR18278052
SRX14415981	SRX14415972	SRR18278062	SRR18278053

Type: Genome, Layout: paired, Platform: Illumina, Format: fastq: Source: RNA, Region Texas, USA, North America.
